# Abdominal wall reconstruction with components separation and mesh reinforcement in complex hernia repair

**DOI:** 10.1186/1471-2482-14-25

**Published:** 2014-04-30

**Authors:** Claire L Nockolds, Jason P Hodde, Paul S Rooney

**Affiliations:** 1 Royal Liverpool Hospital, Prescot Street, Liverpool, Merseyside, L7 8XP, UK; 2 Cook Biotech Incorporated, 1425 Innovation Place, West Lafayette, IN 47906, USA

**Keywords:** Hernia, Contamination, Infection, Components separation, Biologic graft, Mesh, Reinforcement

## Abstract

**Background:**

Abdominal closure in the presence of enterocutaneous fistula, stoma or infection can be challenging. A single-surgeon’s experience of performing components separation abdominal reconstruction and reinforcement with mesh in the difficult abdomen is presented.

**Methods:**

Medical records from patients undergoing components separation and reinforcement with hernia mesh at Royal Liverpool Hospital from 2009 to 2012 were reviewed. Patients were classified by the Ventral Hernia Working Group (VHWG) grading system. Co-morbidities, previous surgeries, specific type of reconstruction technique, discharge date, complications and hernia recurrence were recorded.

**Results:**

Twenty-three patients’ (15 males, 8 females) notes were reviewed. Median age was 57 years (range 20-76 years). Median follow-up at the time of review was 17 months (range 2-48 months). There were 13 grade III hernias and 10 grade IV hernias identified. Synthetic mesh was placed to reinforce the abdomen in 6 patients, cross-linked porcine dermis was used in 3, and a Biodesign^®^ Hernia Graft was placed in 14. Complications included wound infection (13%), superficial wound dehiscence (22%), seroma formation (22%) and stoma complications (9%). To date, hernias have recurred in 3 patients (13%).

**Conclusions:**

Components separation and reinforcement with biological mesh is a successful technique in the grade III and IV abdomen with acceptable rate of recurrence and complications.

## Background

Abdominal reconstruction using components separation in the presence of enterocutaneous fistula, stoma or infection is challenging. Large incisional hernias vary in their complexity and in the past it has been difficult to compare outcomes of the different reconstructive techniques.

In 2010, the Ventral Hernia Working Group (VHWG) devised a grading system to stratify a patient’s risk of developing post-operative complications. Grade III hernias are potentially contaminated due to the presence of stoma, violation of the gastrointestinal tract or previous wound infection, while Grade IV hernias include hernias with a concomitant infected abdomen [1]. Houck et al [2] showed a 41% chance of re-infection and Iqbal et al [3] showed an increase in hernia recurrence after repair of Grade IV hernias. The grading system doesn’t take into account the size or the complexity of the hernia, but it is generally established that complications increase along with the size and complexity of the defect.

The aims for surgery in these complex patients are to initially perform enteroclysis and, if possible, restore intestinal continuity before proceeding to the abdominal wall reconstruction. Different surgical techniques to reconstruct the abdominal midline are required depending on the complexity of the hernia. The repair is then reinforced with mesh [1].

Controversy still exists with regard to abdominal reconstruction, the types of mesh and the positioning of the mesh. We present our experience of abdominal reconstruction with components separation and abdominal wall reinforcement in the Grade III and Grade IV abdomen and hope to clarify some of these issues.

## Methods

### Study design

Approval for this retrospective audit was granted by the techniques and devices committee at Royal Liverpool Hospital where waiver of informed consent was granted. Patient consent was obtained to publish the surgery images contained in this manuscript. All review procedures were conducted according to the principles outlined in the Declaration of Helsinki. Medical records of 23 patients undergoing abdominal wall reconstruction at Royal Liverpool Hospital between 2009 and 2012 were retrospectively analysed. Patient co-morbidities, hernia classification, and previous surgeries were identified. Operative notes were studied and the surgical reconstruction technique was recorded. Length of stay, complications and recurrence were also noted.

### Pre-surgical work-up

**Figure 1:**
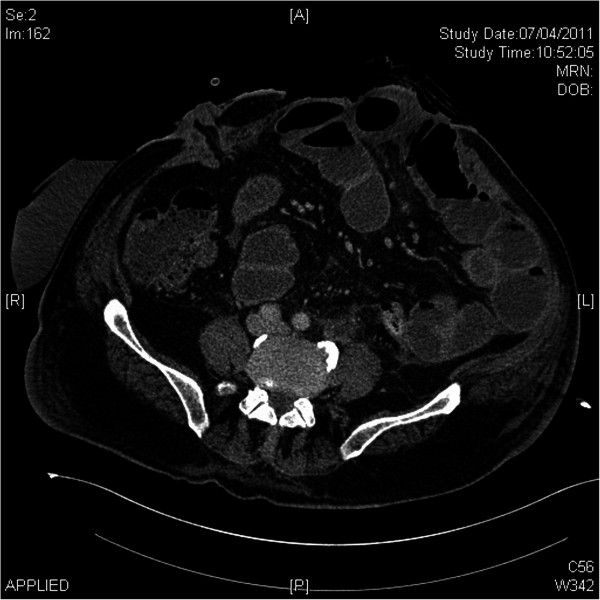
CT scan demonstrating a patient with a stoma and a large incisional hernia with loss of domain.

Preoperatively, all patients were assessed clinically. Their hernia was graded using the Ventral Hernia Working Group system, taking into account co-morbidities, presence of stoma, fistula or infection [1]. For patients presenting with fistulas, contrast studies were performed to confirm the fistula anatomy and a CT scan was obtained to assess the size of the hernia, extent of loss of domain, and to identify occult hernia defects so that the surgical technique could be planned (Figure 1).

### Surgical technique

Laparotomy, adhesiolysis and restoration of intestinal continuity, if achievable, was performed. The abdominal midline was then reconstructed; the Rectus sheath was mobilised and the Ramirez technique of components separation [4] was used to close the midline depending on the individual patient and defect. Finally, the repair was reinforced with mesh with wide overlap [5], with the intent to situate it in a sublay position. In most cases, only one mesh was placed in the position noted (Table 1). When mesh was placed as both a sublay and onlay, a large piece of mesh was placed diagonally as a sublay, the corners were cut, and the cut pieces were used as an onlay over the lateral release of the component separation. Collatamp^®^G sponges were placed in some patients prior to closure as a means to reduce the risk of surgical site infection.

**Table 1:** Position of the mesh

**Type of reconstruction**	**Grade III n = 13**	**Grade IV n = 10**	**Total n = 23**
Onlay mesh	6	7	13
Sublay mesh	3	0	3
Sublay and onlay mesh	3	1	4
Inlay mesh	1	2	3

### Post-operative care

Patients remained in the hospital following their surgery until they were ambulatory and their bladder and bowel functions were normal. Drains were left in for an average of 14 days and removed after discharge. Patients were followed up regularly for the first 3 months and then were followed up on an as-needed basis.

## Results

Twenty-three patients with complex medical histories (Table 2) underwent abdominal reconstruction with mesh reinforcement at Royal Liverpool Hospital between 2009 and 2012. A total of 13 patients presented with Grade III hernias and 10 patients presented with Grade IV hernias. Of these 23 patients, 15 had stomas at the time of presentation. Defect width ranged from 8 cm to 17 cm. Seven of the 10 patients (70%) with Grade IV hernias presented with an enterocutaneous fistula as compared to only one patient with a Grade III hernia. The enterocutaneous fistulas varied in their aetiology and ranged from complications secondary to Crohn’s Disease to 4 patients having post-operative complications arising from appendectomy, post cystoproctectomy ileo-conduit, superior mesenteric artery embolization, or parastomal hernia repair. The median day of discharge was 9 days for Grade III and 16.5 days for Grade IV hernias. Median follow up was 17 months. Baseline demographic information and follow-up information is presented in Table 3.

**Table 2:** Patient medical histories

**Previous operations/medical problems**	**Grade III Hernia (n = 13)**	**Grade IV Hernia (n = 10)**
Pelvic excenteration for anal SCC	2	0
Ileoanal pouch for ulcerative colitis	4	0
Subtotal colectomy for ulcerative colitis	2	3
Hartmans procedure for diverticular disease	1	0
Multiple operations for crohns disease	2	3
Incisional hernia repair	1	0
Laparotomy leading to enterocutaneous fistula (not crohns)	0	4

One patient with a Grade III hernia had multiple previous operations and is not included in the table.

**Table 3:** Results of patients undergoing abdominal wall reconstruction for Grade III and IV incisional hernias

**Results**	**VHWG Grading**		
	**III**	**IV**	**Total**
Number of cases	13	10	23
Median age (min-max)	59 (42-76)	51 (20-76)	57 (20-76)
Male: female	8:5	7:3	15:8
Stoma	8	7	15
Enterocutaneous fistula	1	7	8
Anastomosis	4	5	9
Median discharge day (min-max)	9 (3-70)	16.5 (7-60)	12 (3-70)
Median follow up, months (min-max)	14 (3-46)	18.5 (2-48)	17 (2-48)

**Figure 2:**
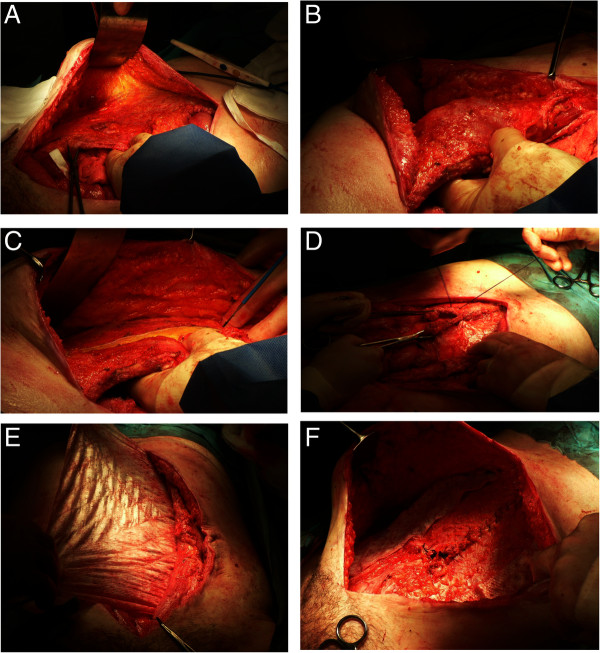
**Ramirez technique of components separation using a sublay and onlay Biodesign mesh on a patient with a Grade 3 incisional hernia. A)** Rectus sheath mobilised. **B)** Rectus sheath now free laterally. **C)** Sheath incised vertically, lateral to semilunaris. **D)** Posterior layer is closed. **E)** A sublay Biodesign graft is sutured in place. **F)** Remnants of graft are sutured over the lateral releasing incisions.

Of all patients studied, 14 (61%) needed components separation using the Ramirez technique (Figure 2) to help achieve midline closure. Rectus sheath mobilisation was performed in all patients to help re-approximate the midline. In 12/13 (92%) of patients with Grade III hernias and 8/10 (80%) of patients with Grade IV hernias the midline closure was achieved. Of the 15 patients who presented with stomas, 4 were reversed at the time of surgery. Of the 11 that were not reversed, 2 underwent pelvic excenteration for anal cancer; the others were complex patients in whom it was either not technically possible to anastomose or due to co-morbidities it was felt the leak rate would have been too high.

An abdominal wall reinforcement material was used to help reinforce the midline closure in all patients. Depending on the size of the defect, a 20×20 cm, 20×30 cm or 30×30 cm mesh was used. Synthetic mesh was placed in 6 patients, while a biologic graft was placed in 17. Details of the different materials used are presented in Table 4, and the different placement positions are described in Table 1. Of note, in the 3 cases where complete closure of the anterior sheath was not possible, the mesh position is described as an inlay. In 7 patients, 4 with Grade III hernias and 3 with Grade 4 hernias, Collatamp^®^G sponges were placed prior to closure as a means to reduce the risk of surgical site infection.

**Table 4:** Types of abdominal wall reconstruction

**Type of reconstruction**	**Grade III n = 13**	**Grade IV n = 10**	**Total n = 23**
Rectus sheath mobilised	13	10	23
Lateral release	7	7	14
Ultrapro prolene mesh	3	0	3
Proceed mesh	1	2	3
Biodesign hernia graft	8	6	14
Crosslinked porcine dermis	1	2	3

Postoperative surgical site complications are shown in Table 5 and included ischaemic stoma in 2 patients and prolonged seroma formation in 5 patients. The cause of the ischaemic stoma complications was thought to be related to a too-tight repair; both of these patients were treated with local refashioning of the stoma after a 3 month period of nutrition and control of wound sepsis.

**Table 5:** Postoperative surgical site complications

	**Biodesign n = 14**	**Synthetic mesh n = 6**	**Crosslinked porcine dermis n = 3**
Seroma	4	1	0
Recurrence	1	1	1
Infection	2	1	0
Wound dehiscence	1	1	3
Ischaemic stoma	1	0	1

**Figure 3:**
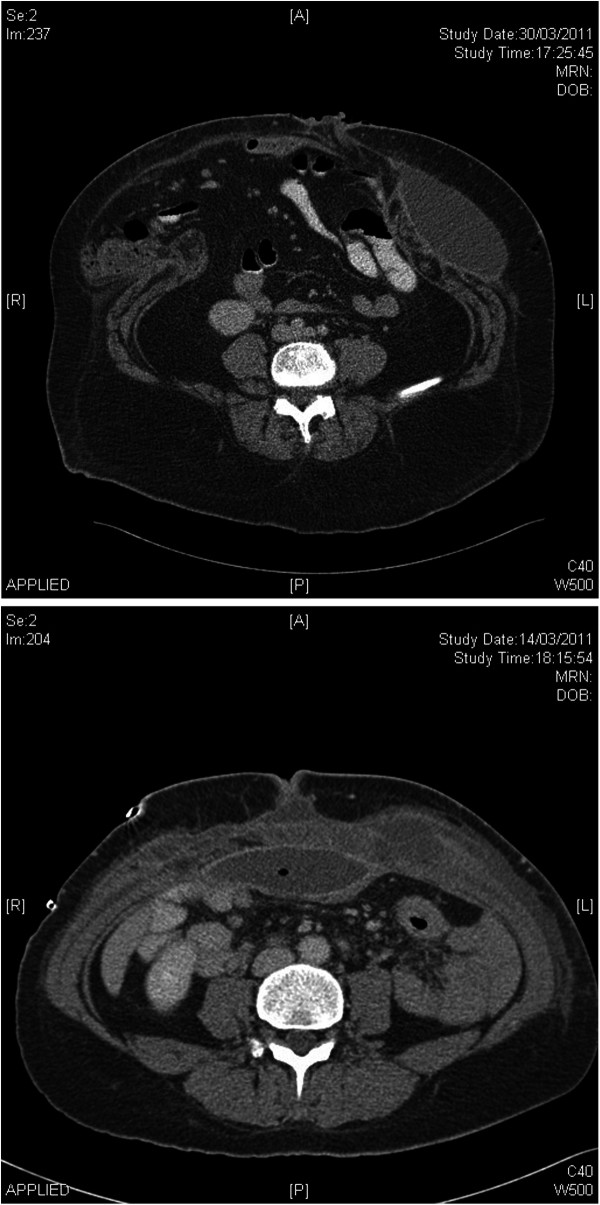
**CT scan images of (i) Top image; complete rupture of an incisional hernia repair; (ii) Bottom image; Seroma formation above and below a sublay mesh repair.**

Figure 3 demonstrates CT scan images of a complete rupture of a hernia repair and seroma formation above and below a sublay mesh. To address the significant number of seromas that form in these patients, we now seek to minimise seroma formation by routinely placing 2 -3 redivac drains above and below the anterior rectus sheath at the time of surgery and leave them for approximately 2 weeks or until the daily fluid output is less than 25 ml.

## Discussion

Abdominal reconstruction in the contaminated or potentially-contaminated abdomen is challenging because of the high risk of re-infection and hernia recurrence. Surgical techniques, synthetic meshes, and biologic graft materials that allow for complete restoration of the midline fascia have been developed to improve outcomes in these patients. For example, in a study comparing suture repair of incisional hernia to mesh repair, it was found that “mesh repair results in a lower recurrence rate and less abdominal pain and does not result in more complications than suture repair” after long term follow-up [6]. In this series, we demonstrate that components separation and midline reinforcement with a variety of graft materials in Grade III and Grade IV hernias is safe and feasible with low morbidity and a low risk of medium-term recurrence.

The VHWG classification was used in this study as we feel it identifies high risk patients and those in which we could consider to use a biological mesh. The European Hernia Society (EHS) have published their own classification which includes the location and size of the hernia defect; however the potential contamination and infection risk is not mentioned [4]. A combination of the VHWG and EHS classification would enable an incisional hernia to be fully described; however in our subgroup of patients, all with large, complex hernias, the emphasis was on the potential of contamination. In 7 patients, we determined that the risk of post-operative surgical site infection was so high that we opted to place Collatamp^®^G, a fully resorbable collagen “sponge” impregnated with high doses of fast-release gentamicin for local delivery of broad spectrum antibiotic, prior to abdominal wall closure.

Reconstituting the midline is an important step in the repair to reduce recurrence; we achieved this in 87% of all patients. A variety of reconstruction techniques have been described in the literature [7,8]. The Ramirez technique is common and successfully allows separation of the abdominal wall components for rectus advancement and achieves closure of the midline in 80% of cases [8]. In larger or complex hernias it is sometimes necessary to adopt double-breasting of the fascia techniques [5] and other methods to gain more width. The importance of achieving midline closure has been demonstrated recently by Itani et al [9] who prospectively followed 80 patients undergoing Grade III or IV hernia repair using components separation and biologic mesh reinforcement and found the recurrence rate was increased if the midline wasn’t closed. Similarly, another study of large incisional hernia repairs using polypropylene mesh showed a recurrence rate of 44% using an inlay bridging technique as compared to only 12% when an underlay reinforcement was used [10]. Reinforcing the reconstruction with mesh has also been shown to reduce recurrence rates [11]; the mesh can be placed as an onlay, sublay or inlay.

In this series, 7 out of 23 patients had the recommended sublay mesh; therefore the majority had midline closure and an onlay mesh. Sublay mesh was described by Rives in 1973 as a retromuscular or preperitoneal mesh [12] and is the preferred position because of reduced wound complications and low recurrence rates [7]. In patients who have undergone multiple surgery it is often not feasible to create space for a sublay mesh. If the midline can be closed, an onlay mesh [13] has been shown to be effective, with an 18.5% recurrence rate at 10 years [14]. We feel the most important step to reduce recurrence is to achieve midline closure regardless of the location of mesh reinforcement.

An inlay mesh or ‘bridging mesh’ is to be avoided if at all possible because serious complications, such as adhesion formation, fistulation and hernia recurrence, have been reported [7,15].

Various mesh types are available and can be classified as either biologic or synthetic. Within these two classifications, meshes can further be classified as either non-absorbable or absorbable, and biologic meshes may also be classified as human-derived or animal-derived. While synthetic surgical meshes have been used successfully for many decades, complications associated with adhesion, erosion, persistent infection, persistent inflammation, fistula formation, seroma formation, and hematoma are common. Additionally, they are relatively contraindicated for use in Grade III and Grade IV hernias due to the risk of chronic infection. For example, Kapiris et al [15] reported 282 cases of seroma and hematoma formation in 3530 hernias (8%) repaired with polypropylene mesh using the transabdominal (TAPP) approach, and Bingener et al [16] noted adhesion formation in 35% of patients following laparoscopic ventral incisional hernias repair with polypropylene mesh.

Initially, we used synthetic mesh in 6 patients but more recently, we have chosen to use biologic graft materials in Grade III and Grade IV hernias due to recommendations made by the VHWG. Biologic mesh materials have been introduced to the market in an attempt to minimize the complications associated with synthetic materials. Examples of biologic surgical meshes include Peri-Guard^®^ (Synovis), Permacol^®^ (Covidien), AlloDerm^®^ and Strattice^®^ (Life Cell), and Biodesign^®^ (Cook). Peri-Guard, Permacol, Strattice and Biodesign products are manufactured from collagen obtained from animal tissues, while AlloDerm is derived from human dermal tissue. The Peri-Guard and Permacol products have been cross-linked using chemical methods to minimize immunogenicity and to make them more resilient in the face of contamination. The Biodesign, Strattice and AlloDerm surgical mesh products are not cross-linked and are often associated with remodelling of new tissues.

While not specifically indicated for use in Grade III and Grade IV hernias, biologic meshes may minimize the adverse events seen with synthetic materials because they more closely recapitulate the natural tissue environment into which they are placed. These natural tissue meshes can be fabricated to integrate quickly with the patient’s tissues, allow rapid angiogenesis to allow the patient to combat infection and stimulate the deposition of additional host connective tissue, optimizing tissue restoration in ways that synthetic mesh materials are unable. Additionally, in the contaminated abdomen, many of the biologic meshes do not have to be removed in the face of infection.

The most common post-operative complication that we experienced in our series with biologic graft materials was transient seroma formation following implant. While most of these seromas are associated with the level of complexity of the dissection and repair, they often resolve, but can cause patient discomfort and impair the healing process. In order to minimize the extent of seroma formation, we now routinely employ the use of 2-3 drains that are left in place above and below the graft reinforcement until daily fluid output is less than 25 ml. A study of 37 patients undergoing repair of enterocutaneous fistula and abdominal reconstruction reported an anastomotic leak in 4 out of 37 and a hernia recurrence rate of 32% [17], in our series there was no anastomotic leaks and a hernia recurrence rate of 12.5% in the enterocutaneous fistula patients (1 out of 8).

The low incidence of recurrence and complications in this series prevents us from clearly assessing the effect of mesh type or location on outcomes. A larger series examining these variables is warranted.

## Conclusion

In this initial experience, utilising components separation and midline reinforcement with a biologic graft material in large, complex Grade III and Grade IV hernias is a safe and feasible alternative to traditional, non-reinforced hernia repair with a minimal recurrence rate and satisfactory results in medium-term follow-up.

## Abbreviations

VHWG: Ventral hernia working group.

## Competing interests

CN has no competing interests. JH is an employee of Cook Biotech. PR has participated as a preceptor and trainer for Cook and has received reimbursement from Cook for his activities. Cook Biotech provided funds to support the publication of this study, but otherwise played no role in the study design or implementation.

## Authors’ contributions

CN collected the data, prepared the manuscript, and contributed to the analysis and interpretation of the results. JH prepared the manuscript and contributed to the analysis and interpretation of the results. PR performed the surgical procedures, collected the data, and contributed to the analysis and interpretation of the results. All authors read and approved the final manuscript.

## Pre-publication history

The pre-publication history for this paper can be accessed here:

http://www.biomedcentral.com/1471-2482/14/25/prepub
